# Unlocking the Therapeutic Potential of *Stevia rebaudiana* Bertoni: A Natural Antiglycating Agent and Non-Toxic Support for HDF Cell Health

**DOI:** 10.3390/molecules28196797

**Published:** 2023-09-25

**Authors:** Rinkey Shahu, Dinesh Kumar, Ahmad Ali, Kanchanlata Tungare, Khalid Mashay Al-Anazi, Mohammad Abul Farah, Renitta Jobby, Pamela Jha

**Affiliations:** 1 Amity Institute of Biotechnology, Amity University Maharashtra, Mumbai–Pune Expressway, Bhatan, Panvel, Mumbai 410206, Maharashtra, Indiarjobby@mum.amity.edu (R.J.); 2 Amity Centre of Excellence in Astrobiology, Amity University Maharashtra, Mumbai–Pune Expressway, Bhatan, Panvel, Mumbai 410206, Maharashtra, India; 3 Department of Life Sciences, University of Mumbai, Vidyanagari, Santacruz (East), Mumbai 400098, Maharashtra, India; dineshkumar@mu.ac.in (D.K.); ahmadali@mu.ac.in (A.A.); 4 School of Biotechnology and Bioinformatics, D. Y. Patil Deemed to Be University, Plot No. 50, Sector 15, CBD Belapur, Navi Mumbai 400614, Maharashtra, India; 5 Department of Zoology, College of Science, King Saud University, P.O. Box 2455, Riyadh 11451, Saudi Arabia; kalanzi@ksu.edu.sa (K.M.A.-A.); mfarah@ksu.edu.sa (M.A.F.); 6 Department of Biological Sciences, Sunandan Divatia School of Science, NMIMS Deemed to Be University, Vile Parle (West), Mumbai 400056, Maharashtra, India

**Keywords:** advanced glycation end product (AGEs), anti-glycation, glucose uptake, protein aggregation, *Stevia rebaudiana*, viability

## Abstract

Sugar carbonyl groups interact with protein amino groups, forming toxic components referred to as advanced glycation end products (AGEs). The glycation system (BSA, a model protein, and fructose) was incubated for five weeks at 37 °C in the presence and absence of Stevia leaf extract. The results indicated that the leaf extract (0.5 mg/mL) decreased the incidence of browning (70.84 ± 0.08%), fructosamine (67.27 ± 0.08%), and carbonyl content (64.04 ± 0.09%). Moreover, we observed an 81 ± 8.49% reduction in total AGEs. The inhibition of individual AGE (argpyrimidine, vesper lysine, and pentosidine) was ~80%. The decrease in the protein aggregation was observed with Congo red (46.88 ± 0.078%) and the Thioflavin T (31.25 ± 1.18%) methods in the presence of Stevia leaf extract. The repercussion of Stevia leaf extract on DNA glycation was examined using agarose gel electrophoresis, wherein the DNA damage was reversed in the presence of 1 mg/mL of leaf extract. When the HDF cell line was treated with 0.5 mg/mL of extract, the viability of cells decreased by only ~20% along with the same cytokine IL-10 production, and glucose uptake decreased by 28 ± 1.90% compared to the control. In conclusion, Stevia extract emerges as a promising natural agent for mitigating glycation-associated challenges, holding potential for novel therapeutic interventions and enhanced management of its related conditions.

## 1. Introduction

The Maillard reaction, or glycation process, binds sugar reactive carbonyl groups to protein amino groups [1], generating harmful advanced glycation end products (AGEs) via Schiff base and Amadori product transformations [2]. These reactions’ intermediates contribute to reactive oxygen species (ROS) generation, impairing antioxidative defenses and disturbing tissue components [3]. ROS and dicarbonyl molecules worsen AGE formation through oxidative and carbonyl stress [4]. These glycoxidation processes have implications beyond diabetes, affecting neurological disorders and cancer [5]. With its pivotal role in diabetic complications and neurodegenerative diseases, glycation research has surged. AGEs activate various mechanisms, driving microvascular and macrovascular diabetic complications [6]. The current emphasis lies in strategies to prevent glycated product production, potentially revolutionizing disease management.

Advancements made over the past decades have propelled the identification of glycated products and the underlying processes stemming from glycation. A spectrum of both qualitative and quantitative methodologies has been developed, illuminating the intricacies of this phenomenon [7]. Traditional glycated materials undergo meticulous examination via diverse techniques, including spectrophotometric and electrophoretic methods [8]. Recent strides have introduced sophisticated tools like spectrofluorimetry, high-performance liquid chromatography (HPLC), and liquid chromatography-mass spectrometry (LC-MS), offering heightened precision in quantifying AGEs and evaluating glycation-related processes [9]. Concurrently, endeavors have been directed towards formulating therapeutic strategies targeting AGE production and its related conditions. Amid these efforts, synthetic agents like aminoguanidine have been deployed, although their utility has been marred by undesirable effects [10]. It is worth noting that certain treatments designed to mitigate hyperglycemia inadvertently trigger weight gain, thereby exacerbating the complexities inherent in managing type 2 diabetes and obesity. These two interconnected conditions synergistically promote insulin resistance via heightened adipogenesis [11,12].

Furthermore, the co-occurrence of diabetes and obesity significantly amplifies the risks of morbidity, mortality, and enduring complications [13]. Recent advances in phytochemistry and analytical techniques have unveiled a treasure trove of natural compounds. Among them, curcumin, eugenol, rutin, garcinol, and various other naturally occurring chemicals have demonstrated remarkable antioxidant and anti-glycation capabilities [14]. Notably, the phenolic phytochemicals inherent in herbs, fruits, and vegetables not only serve as potent antioxidants and glycation inhibitors but also carry a reputation for being generally safe for consumption.

In the span of the past several decades, considerable attention has converged on harnessing the potential of natural compounds for disease prevention and therapeutic intervention. Derived from plants, polyphenols and flavonoids have emerged as noteworthy players in the treatment and mitigation of conditions spanning diabetes, aging, and cardiovascular diseases [15].

Recently, there has been a pronounced emphasis on investigating the potential antiglycation properties of various phytochemicals. Martins et al. [16] conducted a study elucidating the antiglycation activity of *P. Glomerata* fruit hydroethanolic extract against the BSA–Ribose model, utilizing the relative electrophoretic mobility method. Similarly, Rodwattanagul et al. [17] examined the antiglycation potential of the ethanol leaf extract of *Caesalpinia mimosoides* and *Spondia dulcis*, employing the BSA–Ribose model. In parallel, Rodrigues et al. [18] and Fecka et al. [19] explored the antiglycation properties of the ethanol leaf extract of *Varronia curassavica* Jacq. (*Boraginaceae*) and the leaf aqueous extract of peppermint using the BSA–Methylglyoxal model. Among the plethora of reports highlighting plant-based antiglycation activity, Stevia stands as a notable contender. Ali et al. [20] investigated the antiglycation activity within the Morita II variety of *Stevia rebaudiana*, employing the BSA–fructose model to discern its potential impact.

*Stevia rebaudiana*, renowned for its natural sweetness, has garnered attention for a plethora of health benefits including antioxidant, antidiabetic, anti-inflammatory, anti-obesity, antihyperlipidemic, antiviral, anticancer, platelet aggregation inhibitory, hypotensive, and antibacterial activities [21]. This study delves into the potential impact of varied concentrations of Stevia plant extract on the fructose-mediated glycation process.

## 2. Results

### 2.1. Measurement of Browning

**Figure 1:**
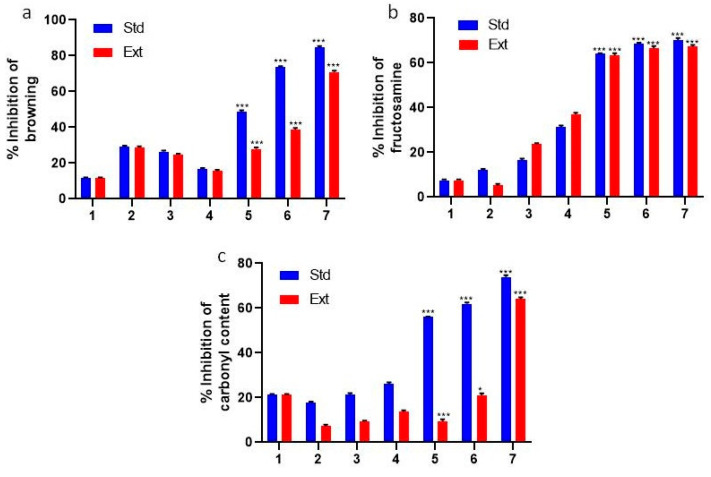
Estimation of (**a**) browning; (**b**) fructosamine; and (**c**) carbonyl content (1: B; 2: B + AG/S − 0.01 mg/mL; 3: B + AG/S − 0.1 mg/mL; 4: B + AG/S − 0.5 mg/mL; 5: B + F + AG/S − 0.01 mg/mL; 6: B + F + AG/S − 0.1 mg/mL; and 7: B + F + AG/S − 0.5 mg/mL). B—BSA; F—fructose; S—extract of *Stevia*. The findings are shown as mean ± SD, with varying levels of significance, * *p* < 0.05, *** *p* < 0.001.

Traditionally, browning has been regarded as a pivotal indicator of the Maillard reaction process [7]. In the context of the fructose-mediated glycation system, Stevia leaf extracts exhibited an inhibitory effect on browning within a concentration range of 0.01 to 0.5 mg/mL. Notably, this inhibition ranged from 27.85 ± 0.09% to 70.84 ± 0.08%. Conversely, in the same glycation system, concentrations of AG spanning from 0.01 to 0.5 mg/mL showcased a reduction in browning, ranging between 48.65 ± 0.01% and 84.68 ± 0.09% upon completion of the fifth week of incubation (Figure 1a).

### 2.2. Determination of Fructosamine

As AGE production escalates, there is a concurrent elevation in the fructosamine and oxo-aldehyde levels [22]. The impact of Stevia leaf extracts (0.01, 0.1, and 0.5 mg/mL) on the fructosamine levels is depicted in Figure 1b. In the fifth week of incubation, the fructose glycation system (positive control) witnessed a substantial augmentation in fructosamine synthesis. In contrast, the presence of Stevia leaf extract exhibited a notable mitigating effect. Specifically, the Stevia leaf extract at a concentration of 0.5 mg/mL led to a remarkable reduction in fructosamine production by 67.27 ± 0.08 (*p* < 0.01). Similar attenuation, measuring 70.22 ± 0.06 (*p* < 0.01), was observed with AG at the same concentration.

### 2.3. Determining the Carbonyl Content of Proteins

To assess the radical scavenging potential, we measured the decay of the stable free radical DNPH, indicating radical recovery facilitated via antioxidants or radical species [23]. The shift from 515 to 370 nm in DNPH intensity reflects radical recovery, transitioning from deep yellow to vivid yellow. Stevia leaf extract (0.01, 0.1, and 0.5 mg/mL) significantly enhanced radical scavenging, leading to increased carbonyl concentration. Intriguingly, while glycated BSA displayed rising carbonyl levels, AG and Stevia leaf extracts demonstrated carbonyl reduction over five weeks of incubation, reaching 73.89 ± 0.47% and 64.04 ± 0.09%, respectively, at 0.5 mg/mL (Figure 1c).

### 2.4. Measurement of Total AGEs and Individual AGEs

**Figure 2:**
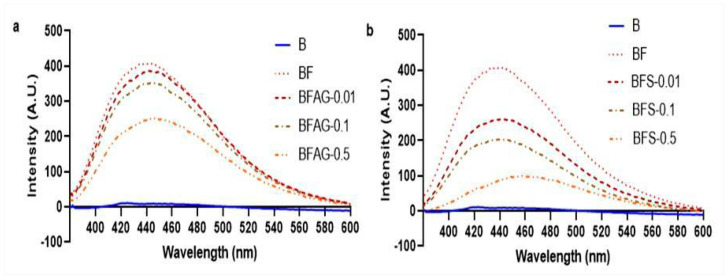
Fluorescent spectra of samples with and without (**a**) AG and (**b**) *Stevia* leaf extract (B—BSA; F—fructose; A—AG; S—extract of *Stevia*).

The process of glycation involving sugars and biomolecules yields AGEs. This investigation utilized BSA and fructose glycation models to explore the potential of Stevia leaf extract as an AGEs’ inhibitor. Evaluating Stevia’s impact at varying doses (0.01, 0.1, and 0.5 mg/mL), the study assessed its capacity to impede total AGEs’ production. Monitoring the progression of fluorescently labeled glycated products, spectrofluorometry was employed, utilizing an excitation wavelength of 370 nm and an emission wavelength at the maximal point of 440 nm [24]. The results demonstrated a reduction of 35.5 ± 3.54% and 81 ± 8.49% in fluorescent AGEs’ formation after the fifth week of incubation. This reduction was evident in the presence of AG and Stevia leaf extract, particularly at the higher concentration of 0.5 mg/mL, within the fructose-mediated glycation system (Figure 2).

**Figure 3:**
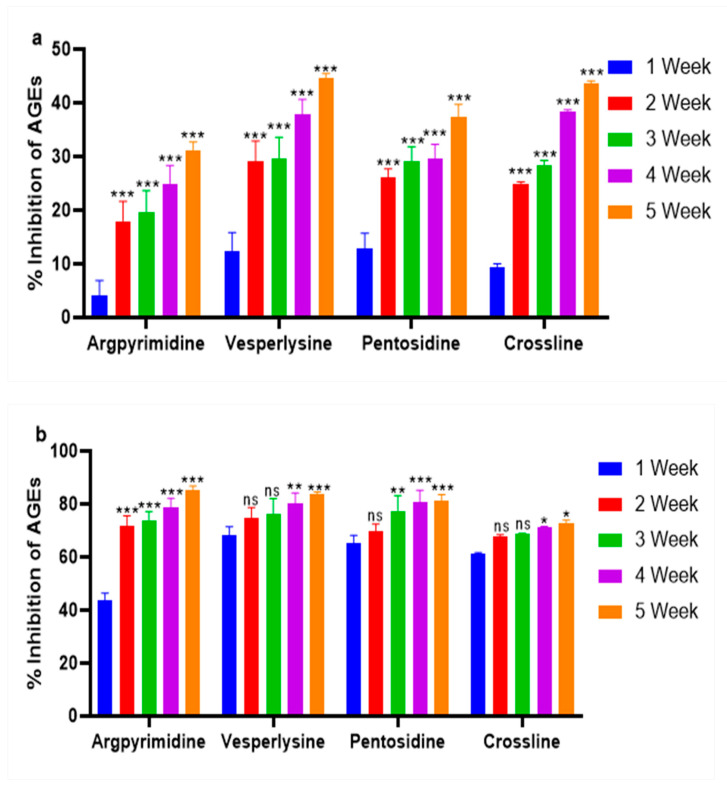
The effect of (**a**) AG and (**b**) *Stevia* leaf extract on the formation of AGEs. The findings are shown as mean ± SD, with varying levels of significance, ns- non-significance; * *p* < 0.05; ** *p* < 0.01; *** *p* < 0.001.

As the glycation process unfolded, distinct types of fluorescent AGEs exhibited varying trends. At the culmination of the five-week incubation period, a notable achievement emerged: the inhibition of vesper lysine formation surpassed that of other fluorescent AGEs, including crossline, pentosidine, and argpyrimidine. This remarkable outcome was particularly pronounced at a Stevia leaf extract concentration of 0.1 mg/mL within the fructose glycation system (Figure 3). Furthermore, a dosage of AG (0.5 mg/mL) demonstrated its effectiveness by reducing the synthesis of argpyrimidine, vesper lysine, and pentosidine by 70%. While the potency of Stevia leaf extract fell short of AG, the elevated concentration (0.5 mg/mL) exhibited promising results. This was highlighted by its ability to curtail argpyrimidine, vesperlysine, and pentosidine synthesis by nearly 80%, along with a 70% reduction in crossline formation (Figure 3).

### 2.5. FTIR Investigation of Amide Bands

**Figure 4:**
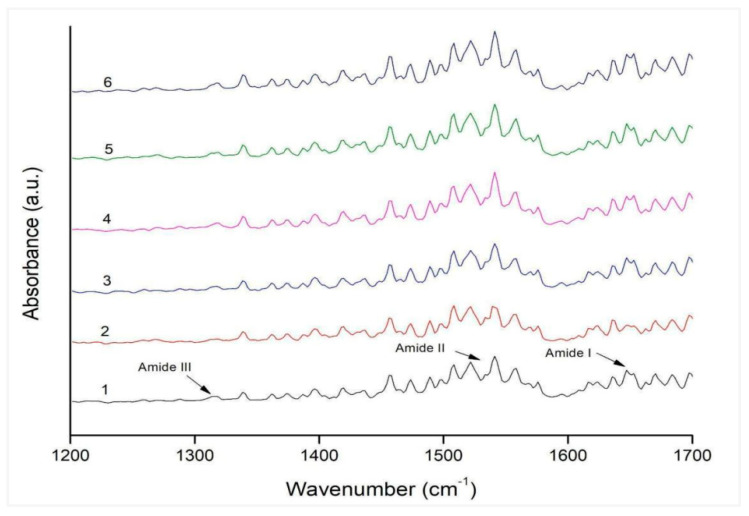
FTIR analysis of the AGEs-specific amide-band. (1: Native BSA; 2: Glycated BSA; 3: Native BSA+ AG; 4: Glycated BSA + AG; 5: Native BSA + *Stevia* extract (0.5 mg/mL); and 6: Glycated BSA + *Stevia* extract (0.5 mg/mL)).

FTIR spectrum analysis serves as a valuable tool for comprehending protein functional group modifications. This study scrutinized the shifting locations of the amide bond, shedding light on structural changes in various samples, including native BSA, glycated BSA, glycated BSA treated with AG at concentrations of 0.01, 0.1, and 0.5 mg/mL, as well as glycated BSA treated with Stevia leaf extract at the same concentrations. Peaks within the amides I (between 1600 and 1700 cm^−1^), II (between 1510 and 1580 cm^−1^), and III (between 1250 and 1350 cm^−1^) regions of the FTIR spectrum provided insight into alterations in the protein’s secondary structure. The distinct vibrations—C=O stretching, in-plane N-H bending, and C-N and C-C stretching are intricately linked to the Amide I, Amide II, and Amide III bands, respectively. In the FTIR spectrum of natural BSA, prominent peaks appeared at 1649 cm^−1^ (Amide I), 1541 cm^−1^ (Amide II), and 1314 cm^−1^ (Amide III). However, in the context of fructose-mediated glycated BSA, these peaks exhibited drastic alterations. Clearly, within the samples treated with AG and Stevia leaf extract (0.5 mg/mL), the functional groups associated with their corresponding amide peaks demonstrated significant resilience, upholding both their functional attributes and structural stability. This conservation also encompassed the protein’s alpha helicity, as depicted in Figure 4.

### 2.6. Determination of Amyloid β-Structure 

**Figure 5:**
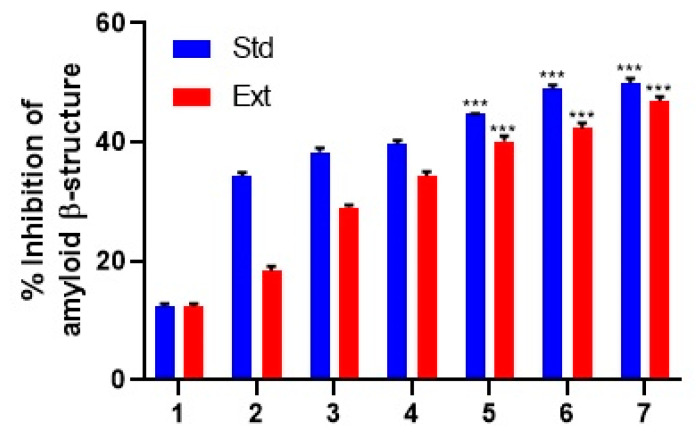
Estimation of protein aggregation via Congo red method (1: B; 2: B + AG/S − 0.01 mg/mL; 3: B + AG/S − 0.1 mg/mL; 4: B + AG/S − 0.5 mg/mL; 5: B + F + AG/S − 0.01 mg/mL; 6: B + F + AG/S − 0.1 mg/mL; and 7: B + F + AG/S − 0.5 mg/mL). B—BSA; F—fructose; S—extract of *Stevia*. The findings are shown as mean ± SD, with level of significance, *** *p* < 0.001.

Glycation triggers the cross-linking and aggregation of proteins, a phenomenon implicated in a range of diseases, including diabetes [25]. To examine protein aggregation, both the Congo red dye method and the thioflavin-*t* test were employed in the presence and absence of Stevia leaf extract (0.01, 0.1, and 0.5 mg/mL). Remarkably, both AG and Stevia leaf extract significantly curtailed protein aggregation to the levels of 49.87 ± 0.09% and 46.88 ± 0.078%, respectively, at a concentration of 0.5 mg/mL, signifying a statistically significant reduction (*p* < 0.01) (Figure 5).

**Figure 6:**
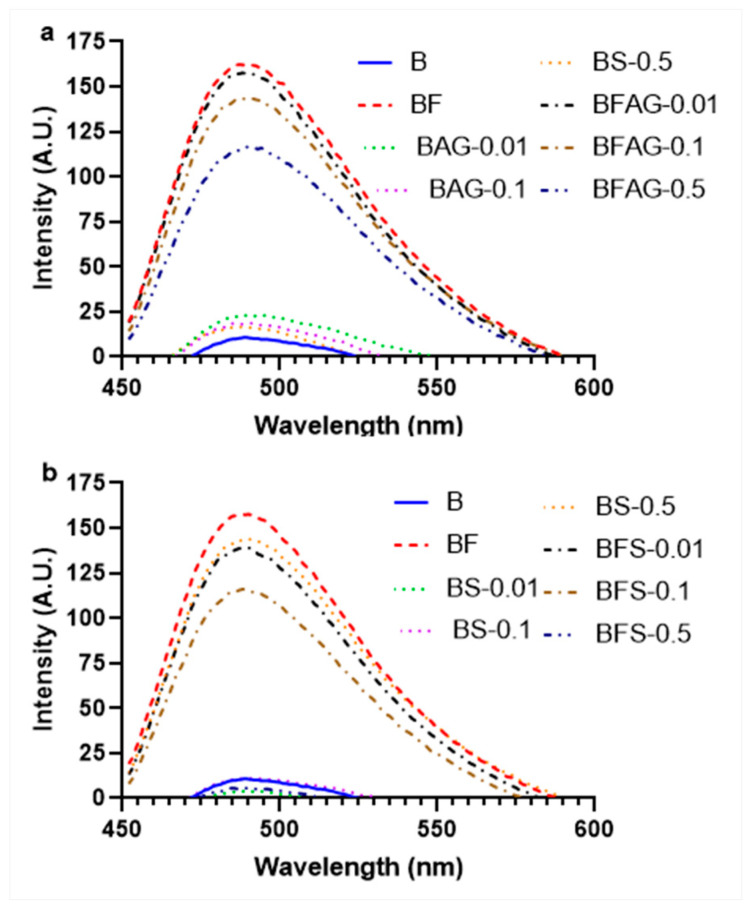
Spectrum of amyloid structure of ThT dye (**a**) AG and (**b**) *Stevia* leaf extract. (B—BSA; F—fructose; A—AG; S—extract of *Stevia*).

Due to its high affinity for amyloid aggregates, the ThT dye serves as a common tool for detecting protein amyloid aggregation. Within our investigation, we sought to evaluate the impact of Stevia leaf extract on the glycated samples, both in the presence and absence of fructose, which is known to foster amyloid structure formation in BSA. This comparison was achieved through the assessment of dye fluorescence intensity. ThT dye exhibits robust binding to the beta-sheet configuration present in amyloid aggregates [26]. Our observations unveiled the highest ThT fluorescence intensity in the glycated albumin samples. Intriguingly, the inclusion of varying concentrations (0.01, 0.1, and 0.5 mg/mL) of Stevia leaf extract effectively mitigated ThT binding and subsequently reduced fluorescent intensity. Within the purview of this study, both AG and Stevia leaf extract demonstrated the ability to curtail β-amyloid aggregation. At a concentration of 0.5 mg/mL, AG exhibited an 18.75% reduction in β-amyloid aggregation, while Stevia leaf extract showcased an even more remarkable 31.25% reduction (Figure 6).

### 2.7. In Vitro DNA Damage Analysis

**Figure 7:**
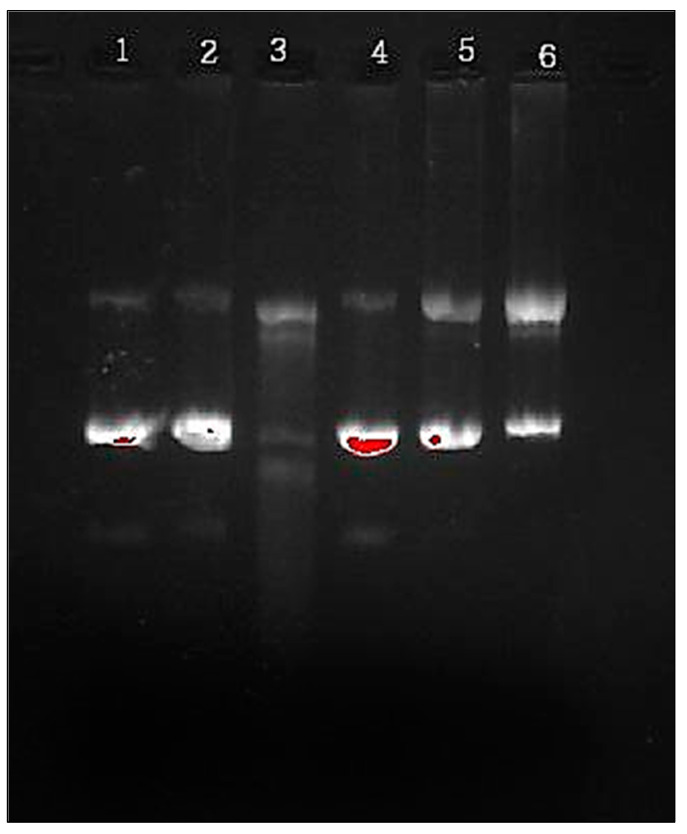
In-vitro DNA damage reversal in presence of *Stevia.* Lane 1—DNA (0.5 μg); Lane 2—DNA (0.5 μg) + *Stevia* (5 μg); Lane 3—DNA (0.5 μg) + Lys (20 mM) + MG (20 mM); Lane 4—DNA (0.5 μg) + Lys (20 mM) + MG (20 mM) + *Stevia* (1 mg/mL); Lane 5—DNA (0.5 μg) + Lys (20 mM) + MG (20 mM) + *Stevia* (0.5 mg/mL); and Lane 6—DNA (0.5 μg) + Lys (20 mM) + MG (20 mM) +*Stevia* (0.1 mg/mL).

Maillard reactions profoundly impact the structural integrity of genomic DNA, revealing a potential correlation between the interaction of lysine and MG and the fragmentation of DNA strands. In the glycated system, DNA undergoes transitions from an initial form to a supercoiled state after incubation with Fe^3+^, eventually adopting a circular form. We further explored the impact of Stevia leaf extract concentrations (ranging from 0.1–1 mg/mL) on DNA strand integrity. At 0.5 mg/mL, the extract reversed DNA damage by approximately 50% (Lane-5). At 1 mg/mL, the extract exhibited a complete restoration of the fragmented DNA strand (Lane-4), as depicted in Figure 7.

### 2.8. 3-[4,5-Dimethylthiazol-2-yl]-2, 5 Diphenyltetrazolium Bromide Assay (MTT ASSAY)

**Figure 8:**
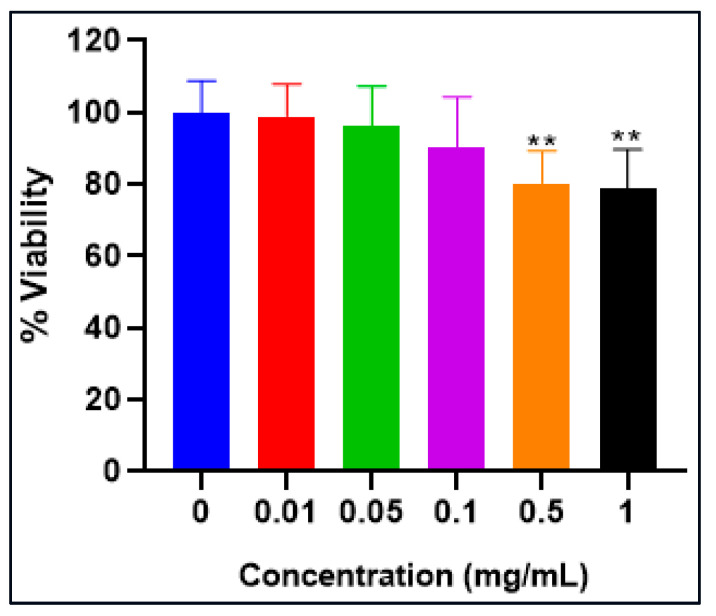
% Viability of HDF cell line after 24 h incubation with combined aqueous extract of *Stevia* using MTT assay. The findings are shown as mean ± SD, with level of significance ** *p* < 0.01.

**Figure 9:**
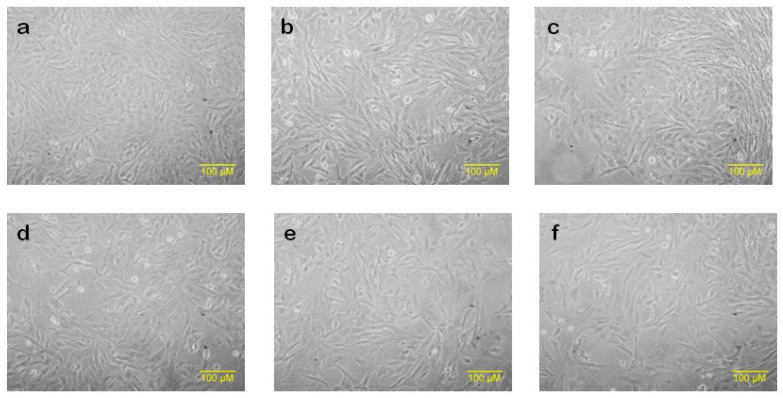
Morphology of HDF cells after the treatment with *Stevia* aqueous extract ((**a**): control; (**b**): 0.01 mg/mL; (**c**): 0.05 mg/mL; (**d**): 0.1 mg/mL; (**e**): 0.5 mg/mL; and (**f**): 1 mg/mL).

Utilizing the MTT test, we delved into evaluating the potential impact of Stevia extract, a process that hinges on assessing mitochondrial metabolism and its implications [27]. Ranging from 0.01 to 1 mg/mL, the aqueous Stevia extract was introduced to the HDF cell line to discern its effects on cellular viability. The results, as illustrated in Figure 8, revealed a gradual decline in HDF cell viability in correspondence with the administered doses. Cells treated with 0.5 and 1 mg/mL of the extract showcased a viability of roughly 80%, juxtaposed against the control group (*p* > 0.01). Strikingly, the cells retained their distinctive fibroblastic attributes, maintaining their characteristic spindle-shaped morphology (Figure 9). In essence, the presence of Stevia extract had a marginal bearing on the viability and morphology of the HDF cell line.

### 2.9. Production of Cytokine

**Figure 10:**
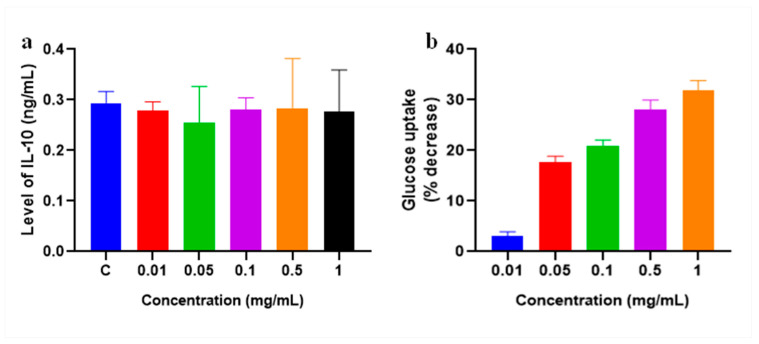
Effect of *Stevia* extract on production of (**a**) anti-inflammatory cytokine IL-10 and (**b**) glucose uptake in HDF cell.

The protective anti-inflammatory response, crucial for mitigating damage, is significantly hindered by glycation [28]. In our study, we examined the impact of Stevia plant extract on IL-10, a key anti-inflammatory cytokine. Despite exposing cells to varying Stevia extract concentrations, we observed only a minor ~5% reduction in the IL-10 levels (Figure 10a). Our findings suggest that the introduction of Stevia extract does not appear to impact the levels of the anti-inflammatory cytokine IL-10. This implies that the defensive mechanism responsible for managing ROS generated within the cell, often compromised by glycation, might remain unaffected.

### 2.10. Glucose Uptake Assay

Glucose serves as the primary cellular energy source, necessitating a continuous supply to cells [29]. Stevia extracts, recognized as a robust, calorie-free sugar substitute, prompted an inquiry into their impact on glucose transport within HDF cells. Following a 24 h incubation period, the escalating dosages of Stevia extract correlated with a decline in glucose uptake (Figure 10b). Exposure to the 0.5 and 1 mg/mL extracts led to reductions of 28% and 30%, respectively, in glucose uptake. This observed decrease might be attributed to the higher presence of diterpene glycosides found within Stevia [30].

In summary, our study underscores the influence of Stevia extract on both the early and intermediate phases of glycation, revealing inhibition activity comparable to that of AG. Notably, in the advanced glycation stage, Stevia exhibited even greater inhibition compared to AG, as indicated in Table 1. This highlights the potential of Stevia extract to effectively intervene at different stages of glycation, and particularly in the later stages, presenting a promising avenue for further exploration and application.

## 3. Discussion

The consequences of the Maillard reaction’s impact on protein by-products contribute significantly to the escalation of diabetic complications. Glucose, a fundamental contributor to diabetes, plays a central role in this context. This study investigated the potential of Stevia leaf extract to counteract the detrimental effects of glycation. Via a fructose-mediated glycation system, the gradual escalation of Stevia leaf extract concentration was observed to result in the inhibition of browning intensity. This attenuation of browning appears to be associated with a reduction in the formation of glycated products. Interestingly, similar inhibitory effects on browning caused by the other plant extracts have been reported. For example, Rubab et al. [31] found that Nigella sativa (at 0.16 mg/mL) reduced protein glycation-induced browning by 47.96%. Our prior research also indicated a 36.95% reduction in browning using Stevia extract (at 1 mg/mL) compared to the positive control [20]. Similarly, Eruygur et al. [32] demonstrated a 54.4% decrease in the browning of BSA in a fructose environment using the *G. cordifolium* leaves’ aqueous extract (at 1 mg/mL).

Fructosamine, the unstable Schiff base complexes formed in the initial stages of glycation, serves as a marker for regulating the blood sugar levels in individuals with diabetes [33]. In this study, Stevia demonstrated remarkable efficacy in reducing the levels of glycated products during the quantification of early glycation outcomes. This effect was comparable to, if not equal to, the standard AG. Specifically, AG exhibited inhibition rates of 45–48% at a concentration of 1 mg/mL. Similar impactful results have been observed in related studies. Nunthanawanich et al. [34] and Perera et al. [35] reported that the leaf extracts of *Moringa oleifera* and *Costus speciosus* at concentrations of 1 mg/mL and 5 mg/mL led to reductions in the fructosamine levels by 49.56% and 10.05%, respectively. Conversely, fructose emerged as a more rapid reducing agent, effectively preventing the formation of diverse Amadori and cross-linking products, protein-bound fluorescence, and its related compounds [36]. Martins et al. [16] also reported analogous glycation inhibition effects in *P. glomerata* hydroethanol extract and aminoguanidine, as determined via relative electrophoretic mobility.

Whenever early glycation products undergo alteration, carbonyl compounds are generated. Research has shown that red grape skin extract, when employed at concentrations of 0.06–0.50 mg/mL, led to a substantial decrease in carbonyl content by 37.7% to 41.7% [37]. In the context of this study, Stevia extract exhibited notable inhibitory effects, even at lower albumin concentrations and doses below 1 mg/mL. By curbing the synthesis of Amadori products and impeding the oxidative degradation of sugars catalyzed by metals, prior studies have demonstrated that antiglycation agents can effectively delay the formation of AGEs [38]. Stevia leaf extract showcases potential in reducing albumin oxidation when incubated with fructose and BSA, primarily by reducing the generation of carbonyl groups. This property enhances its standing as a potent glycation inhibitor. The generation of carbonyl derivatives resulting from amino acid residue oxidation during glycation and glycoxidation processes triggers the production of reactive oxygen species. This, in turn, compromises the protein oxidative defense by depleting thiol groups, thereby leading to cellular protein damage [39]. In the early stages of the glycation process, the formation of a superoxide anion occurs, as the Schiff’s base undergoes 1,2,3-enolization and the enolate anion is subsequently oxidized [40]. The proposed mechanisms underlying the antiglycation action encompass the disruption of intra- and intermolecular cross-linkages, the hindrance of reducing sugar, Schiff base, or Amadori compound formation containing carbonyl or dicarbonyl groups, and the eventual blockade of oxidation and nonoxidative breakdown processes involving late-stage Amadori products [41]. Rodrigues et al. [18] reported a glycation inhibition activity of 61.7% and 60.8% in the ethanolic leaf extract of *Varronia curassavica Jacq*. (*Boraginaceae*) and *Brickellin* at concentrations of 250 µg/mL and 0.125 mM, respectively, while AG at 1 mM exhibited an activity of 70.7% against glycation.

The multi-step glycation process generates ROS and dicarbonyl compounds that impede antioxidant defense systems to varying degrees, resulting in elevated AGEs’ production [42]. AGEs encompass a diverse range of molecules, classified based on features such as fluorescence and cross-linking. Fluorescence techniques are commonly employed for the AGEs’ measurement, and total AGEs were quantified via absorbance within the range of 380–600 nm. Notably, fluorescence in samples with Stevia extract exhibited a reduction. In a different study, 100 μg/mL of *S. alpina* extracts demonstrated over 70% inhibition of protein glycation [6]. Similarly, red grape skin extract and AG at a concentration of 0.5 mg/mL diminished AGEs’ generation by 63.52% and 73.3%, respectively [37]. Escutia-Lopez et al. [43] observed a remarkable 96.5% slowdown in AGEs’ production via the utilization of a 100 mg/mL aqueous extract of Stevia. 

Research focusing on protein glycation has gained prominence, with detailed descriptions of various AGEs’ fluorescence characteristics. In our investigation, Stevia extract significantly lowered the fluorescence of distinct AGEs, including Argpyrimidine, Pentosidine, Vesperlysine, and Crossline. These distinct types of AGEs contribute differently to bodily development and complications. Awasthi and Saraswathi [44] reported reduced levels of numerous AGEs when subjected to varying concentrations of silybin (50, 100, and 500 μM).

Protein aggregates, a consequence of glycation-induced structural alterations, emerge in the later stages of nonenzymatic glycation and are employed to gauge the extent of secondary structural changes within proteins [45]. Various methods, including Congo red and Thioflavin T (ThT), are employed to measure aggregation. In our study, a reversal of fructose-induced BSA aggregation was observed in the presence of Stevia extracts. Adisakwattana et al. [46] reported that *Mesona chinensis* extract, exposed at doses spanning from 0.25 to 1 mg/mL, exhibited a dose-dependent reduction in protein aggregation in the Congo red test. In terms of ThT analysis, Stevia leaf extract demonstrated its potential in mitigating glycation-mediated albumin aggregation. Alvi et al. [47] indicated that glycyrrhizic acid at 50 μM reduced ThT binding by 80.13%, while AG inhibited ThT binding by 71.13%. Pawlukianiec et al. [48] noted a 27% decrease in ThT content in the presence of meloxicam compared to the glycated systems. The ethanolic leaf extracts of *Caesalpinia mimosoides* and *Spondias dulcis* demonstrated antiglycation activities of 11.4 ± 1.1% and 11.5 ± 0.5%, respectively, while the standard aminoguanidine displayed an activity of 3.1 ± 0.7% at 0.1 mg/mL [17]. 

A compelling indicator of the impact of glycation and free radicals on single-strand breaks is evident in the damage inflicted upon pBR322, a DNA molecule. Suji and Sivakami [49] have established that ferric ions (Fe^3+^) and free radicals significantly contribute to DNA strand damage. Their work further highlights that the modification of albumin induced via methylglyoxal (MG) led to the generation of hydrogen peroxide (H_2_O_2_), the formation of MG radical anions, and the production of cross-linked MG radical cations, collectively resulting in DNA strand damage. Kumar and Ali [50] presented findings indicating the reversal of DNA damage via thymoquinone at a concentration of 10 μM. Within our study, Stevia extracts exhibited the capacity to reverse DNA damage in the glycation system involving lysine, MG, and FeCl_3_. This observed DNA protective effect could potentially be a contributing factor to the inhibition of free radical production associated with Stevia extract treatment.

A substantial body of research has highlighted the potential of polyphenols to disrupt the cross-linking structure of AGEs and limit the carbonyl group interactions with lower sugars [41]. These findings corroborate our theory that the potent phytochemical profile of Stevia leaf extract could indeed contribute to the inhibition of AGE production. Mass spectrometry investigations of Stevia have further confirmed the pivotal role of its polyphenolic components in reducing glycation activity [51]

In a recent study, Mahapatra and Bharti [52] proposed a range of strategies for preventing glycation. These include the modification of free amino groups with inhibitors (type A) to thwart sugar binding or the use of supplementary inhibitors (type B) to divert sugars from the Maillard process. Certain inhibitors (type C) capture unbound metal ions to hinder glycoxidation, while others serve as antioxidants. Inhibitors of type D, for instance, impede the intermediate phase of glycation by intercepting reactive dicarbonyls. Few inhibitors (type E) target Amadori products, and an even smaller subset (type F) break down formed AGE cross-links. In this context, Stevia functions as a type C inhibitor by effectively sequestering free ions, thus preventing glycoxidation through its antioxidant properties.

Concerning its potential toxicity to humans, extensive research indicates negligible adverse effects at higher concentrations of Stevia, rendering it safe for use within specified concentrations where it also exhibits its efficacy as an antiglycation agent. This underscores the compound’s promising dual attributes of effectiveness and safety.

In line with our findings, Stevia extract demonstrated no adverse effects on the HDF cell line, reinforcing its non-toxic nature. It is worth noting that various studies have produced differing results concerning the impact of Stevia extract on cell viability. For instance, Jayaraman et al. [53] observed a negligible decrease in Vero cell viability upon exposure to the acetone extract of the Stevia leaf. Rizzo et al. [54] reported 80% viability of HL-60 cells when treated with 5 μM of Stevia extract. Conversely, Kulkarni et al. [55] noted a significant increase in cell survival by 300% in the RAW cell line. In a recent study, Hanna et al. [56] reported 100% cell viability in WI-38 cells when exposed to a hydromethanolic extract of *Stevia rebaudiana* at 500 μg/mL. These diverse findings collectively reflect the variability in Stevia extract’s effects on different cell lines. Importantly, our study’s results are in coherence with the observed data, further strengthening the notion of Stevia extract’s benign impact on cell viability, particularly in the context of HDF cells.

The presence of Stevia extract appears to function as an anti-inflammatory agent, possibly contributing to the modulation of IL-10 activity and the consequent reduction in its levels. This effect suggests that Stevia extract could potentially prevent unwarranted increases in the IL-10 levels, thereby promoting controlled cellular inflammation. The current findings imply that Stevia extract holds promise for possessing anti-inflammatory properties, inviting further exploration in subsequent studies. In a similar vein, Farid et al. [57] documented a reduction in the IL-10 levels by 1.49-fold through the use of 40.5 mg/mL of Stevia extract. 

Our study also delved into the impact of glucose uptake by the cell lines. Notably, previous research has established that stevioside, a component of Stevia, can regulate the blood sugar levels in diabetic rat models by enhancing insulin production and curbing the expression of the PEPCK gene [30]. The PEPCK protein catalyzes the conversion of oxaloacetate into carbon dioxide and phosphoenol pyruvate, thereby stimulating the gluconeogenesis pathway [58]. Consequently, the inhibition of the PEPCK enzyme or reduction in its gene expression would lead to a decrease in glucose synthesis from non-sugar sources [59]. Earlier investigations by Mohd-Radzman et al. [60] and Bhasker et al. [61] involved the use of the 30 µM and 100 µM Stevia extracts on 3T3-L1 cells, respectively, resulting in the reported changes of 2.1-fold and 1.28-fold, respectively. Furthermore, Elnaga et al. [62] observed a 1.25-fold decrease in the glucose levels at a dose of 25 mg/kg body weight in female rats. Minor variations in absolute values across different studies involving Stevia could potentially be attributed to diverse climatic, ecological, and geographic conditions under which the plants were cultivated, thereby contributing to these fluctuations in the reported fold changes [63].

The striking alignment between the outcomes observed in our study and the existing literature strongly underscores the potential of Stevia as a potent antiglycating agent. This congruence across the cell line and in vitro results substantiates Stevia’s promise for effectively managing glycation-related disorders. The harmony between our findings and the established knowledge amplifies the significance of Stevia’s potential therapeutic role, bolstering its prospects for application in the prevention and management of the conditions linked to glycation.

## 4. Materials and Methods

### 4.1. Material

Bovine serum albumin (BSA), Lysine, Methylglyoxal (MG), and Aminoguanidine (AG) were procured from Sigma-Aldrich (St. Louis, MO, USA), and Thermo Fisher Scientific Ltd. (Waltham, MA, USA) supplied the plasmid pBR322. Sodium azide, fructose, 2,4-Dinitrophenylhydrazine (DNPH), Nitro-blue tetrazolium (NBT), Trichloroacetic acid (TCA), and Thioflavin-T (ThT) were purchased from SRL Pvt. Ltd. Particularly, analytical-grade chemicals and reagents were used in the investigations. The National Centre for Cell Science in Pune, India, supplied the HDF cell line. Dulbecco’s Modified Eagle Medium (DMEM), fetal bovine serum (FBS), antibiotics, and formazan were acquired from Mumbai, India’s HiMedia Pvt. Ltd. (Telangana, India). Glucose was purchased from SRL Pvt. Ltd. (Mumbai, Maharashtra, India). The Human Interleukin 10 ELISA Kit was purchased from ImmuoTag, Overland, MO, USA. The GOD/POD kit was purchased from Prietest™ R-Sys, Mumbai, Maharashtra, India.

#### 4.1.1. Plant Material and Identification

Jamuna Biotech Farm in Pune, India, provided the *Stevia rebaudiana* Bertoni variety of SA-178 plants. Dr. Rajendra D. Shinde, a taxonomist at the Blatter Herbarium at St. Xavier’s College in Mumbai, India, recognized the *Stevia rebaudiana* plant (Herbarium specimen no. PJ-1). At the age of three months, a sample of *Stevia* leaves was obtained from the same batch. Plant care was conducted in a greenhouse environment. Plant leaves were rinsed with water for 7–8 days of air drying at 28 ± 2 °C, then crushed and stored as powder.

#### 4.1.2. Extraction of Stevia Plant Leaves

The methodology employed in our study involved the preparation of an aqueous extract of dried leaves, which was modified from the procedure outlined by Woelwer-Rieck et al. [64]. Specifically, a uniform particle-size powder weighing 3 g was blended in 50 mL of distilled water, and the resulting mixture was subjected to vortexing for 1 h in a water bath at 100 °C. Subsequently, the mixture was centrifuged at 4500 rpm for 15 min, and the Whatman No. 1 filter paper was used to collect and filter the supernatant into a 100 mL conical flask. The resulting filtrate was stored at 0–4 °C for further use.

### 4.2. Antiglycation Assays

#### 4.2.1. Albumin Glycation

The antiglycation efficacy of the leaf extract of *Stevia rebaudiana* was evaluated using a model protein, BSA, and a modified fructose glycation system [32]. The bovine serum albumin (BSA) solution was incubated at 10 mg/mL with fructose at 100 mg/mL for five weeks. The incubation was conducted at 37 °C in a 0.1 M phosphate buffer with a pH of 7.4 and contained 3 mM of sodium azide (NaN_3_). The experiment was carried out in the presence or absence of an aqueous extract of *Stevia* leaf at concentrations of 0.01, 0.1, and 0.5 mg/mL. The reaction mixtures were dialyzed using a phosphate buffer with a concentration of 20 mM. Before analysis, all samples that underwent dialysis were stored at a temperature of −20 °C.

#### 4.2.2. Determining the Browning Level

The amount of browning that has occurred is caused by the Maillard reaction at sequential stages of the glycation system. The absorbance of the glycated samples was determined using 420 nm by Kumar and Ali [50].

#### 4.2.3. Measurement of Fructosamine Content

The NBT assay was used to determine the fructosamine content, with minor changes to the method mentioned by Banan and Ali [7]. An amount of 10 µL of the glycated sample and 0.5 mM of the NBT reagent (100 µL) were treated for 15 min at 25 ± 2 °C in a 0.1 M sodium carbonate buffer (pH 10.4). A spectrophotometer was used to measure the absorbance at 530 nm.

#### 4.2.4. Determination of Carbonyl Content

The DNPH approach described by Meeprom et al. [65] assessed the carbonyl content of proteins with a few minor adjustments. Each 100 μL sample was mixed using 10 mM of DNPH in 400 μL of 2.5 M HCl before being left to sit in the dark at 28 ± 2 °C for an hour. The combination was then incubated for 5 min on ice with 0.5 μL of 20% TCA (*w*/*v*) added before being centrifuged for 10 min at 4 °C at 10,000 rpm. The supernatant was removed, and the pellet was rinsed thrice in 500 μL of the ethanol–ethyl acetate (1:1 *v*/*v*) mixture. A solution of 6 M guanidine hydrochloride in 100 μL was used to dissolve the pellet residue. At 370 nm, the absorbance was calculated compared to a blank.

### 4.3. Measurement of Total AGEs and Individual AGEs

The individual and total AGEs were quantified as per the method of Awasthi and Saraswathi et al. [44]. Differences in emission intensity were seen for each sample between the wavelengths of 380 and 600 nm using a spectrofluorometer (Varian, Cary Eclipse model).

Table 2 excitation and emission wavelengths. The reported percentage of inhibition of glycation is shown below:

$$\mathrm{Glycation}\mathrm{inhibition}\;(\%)\;=\frac{\;F.\;I.\;of\;\left(Positive\;control-Test\;Sample\right)}{F.\;I.\;of\;Positive\;control}\times 100$$

### 4.4. FTIR Study on AGE-Specific Amide Bands

The FTIR analysis of the native and fructose-mediated glycation of BSA with and without the presence of Aminoguanidine/*Stevia* leaf extract was analyzed for characteristic functional groups in the glycated protein samples [67]. A spectrophotometer (Perkin Elmer, Inc., Waltham, MA, USA) was used for the FTIR analysis of these materials in the diminutive total reflection mode, with a resolution of 4 cm^−1^ and a spectrum range of 4000–400 cm^−1^ [68].

### 4.5. Determination of Amyloid β-Structure

The evaluation of Congo red’s binding to the amyloid aggregate was performed as per the reports of Bouma et al. [69]. In this experiment, a solution containing 100 μM of Congo red (50 μL) was prepared by mixing it with 10% ethanol/PBS (*v*/*v*). The resulting solution was then incubated with 50 μL of each sample for a period of 20 min at 25 ± 2 °C. The absorbance intensity was quantified at a wavelength of 530 nm. A marker for the β-amyloid cross structure, Thioflavin-T (ThT), was also used to confirm the β-amyloid aggregation [70]. The glycated samples (3 mL) were combined with 20 μM of ThT and then incubated for 1 h. The fluorescence spectrum was then captured between a 440 nm excitation and an emission range of 490–600 nm on a slit-width of 5 nm.

### 4.6. In Vitro Glycation of Plasmid DNA

The Kumar and Ali [50] method was used to investigate the effect of glycation on the integrity of DNA. For the positive control, the plasmid pBR322 alone was also incubated. These samples underwent incubation for 3 h at 37 ± 1 °C. When the incubation period was over, the samples were placed onto 1% agarose gels for 120 V electrophoresis. The gel was analyzed in Gel Doc (Biorad Gel Doc^TM^ XR+), Bio-Rad, Mumbai, India.

### 4.7. MTT Assay

The antiproliferative effects of *Stevia rebaudiana* Bertoni’s aqueous extract were assessed using the HDF cell line via the MTT assay. The cells (4000 per well) were cultured for 24 h in 96-well plates with DMEM supplemented with FBS and penicillin/streptomycin. They were then exposed to the Stevia extract concentrations (0.01 to 1 mg/mL) for 24 h. The MTT solution (0.5 mg/mL) was added, followed by a 4 h incubation at 37 °C with 5% CO_2_, in the dark. Formazan crystals generated by cell metabolism were solubilized in SDS. Absorbance was measured at 570 nm using a microplate reader [71]. Cytotoxicity effectiveness was determined as a percentage of the growth inhibition using the provided formula.

(1) $$\%\;Viability=\frac{\;Absorbance\;of\;test}{Absorbance\;of\;control}\times 100$$

### 4.8. Phase Contrast Microscopy

HDF cells (1 × 10^5^ cells/mL) were cultured in 96-well plates with 5 mL of DMEM containing 10% FBS, 1% penicillin/streptomycin, with 37 °C at 5% CO_2_ for 24 h to monitor the cell line under a phase contrast microscope. After that, the cells were exposed to various concentrations of *Stevia* extract (0.01, 0.05, 0.1, 0.5, 1, 5, 10, 20, and 30 mg/mL) for 24 h. Subsequently, 20 μL of the MTT solution at a concentration of 0.5 mg/mL was injected into each well. The resulting mixture was then incubated for 4 h at 37 °C in an environment containing 5% CO_2_ while kept in the absence of light. Pictures were obtained after 24 h using a Nikon Eclipse TS 100 microscope with a 45X phase contrast lens.

### 4.9. Cytokine Production

This study aimed to determine the IL-10 levels and evaluate *Stevia rebaudiana*’s impact on them. The procedure occurred at room temperature under standard lab conditions. The sample (40 μL) and reference (50 μL) were added to separate wells. Streptavidin-HRP (50 μL) was introduced to all wells except the blank. The anti-IL-10 antibody (10 μL) was added to the sample wells. After mixing, the plate was sealed and incubated at 37 °C for 60 min. The plate underwent five wash cycles with a wash buffer, and substrate solutions A and B (50 μL each) were added to every well. Incubation at 37 °C for 10 min followed, then a stop solution (50 μL) was added, causing a color change. Optical density (OD value) was measured at 450 nm using a microplate reader within 10 min after the stop solution addition.

### 4.10. Glucose Uptake Assay

Glucose absorption in the HDF cells followed the protocol outlined by van de Venter et al. [72]. In a 96-well plate, 6000 cells were exposed to the extract concentrations ranging from 0 to 1 mg/mL. Control wells received no treatment. Subsequently, each well received 3 mM of glucose in DMEM with 10% FCS. After a 24 h incubation, the glucose levels were measured using the GOD/POD method with the prietest touchTM biochemistry analyzer.

### 4.11. Statistical Analysis

The GraphPad Prism (version 8) was used for statistical analysis. The results from three separate experiments, each conducted in triplicate, were presented as mean ± SD. To determine the significance, the one-way analysis of variance (ANOVA) was employed. Tukey’s multiple comparison tests were applied to ascertain treatment differences, denoted as *p*-values of 0.05 *, 0.01 **, and 0.001 ***.

## 5. Conclusions

Plant extracts can reduce AGE-induced problems in diabetes due to their antiglycation properties. The plant-derived bioactive antiglycation substances could be beneficial in developing next-generation therapies to combat diabetic complications and prevent aging. It also emphasizes the significance of an antioxidant-rich diet as a component of an all-encompassing diabetes treatment strategy. Stevia’s aqueous leaf extract strongly inhibits the Maillard reaction’s formation, protein denaturation, and protein cross-linking. According to these findings, Stevia leaf extract may be utilized as an antiglycation and anti-aggregation agent to stop or delay the onset of diabetes-related problems. The study also showed that Stevia extract can inhibit glucose uptake via HDF cells without causing any harm to the cells. The sparing effect of Stevia extract on the IL-10 levels may point to its role in modulating immune responses and inflammation, fostering tissue repair and wound healing, and reducing autoimmune diseases and chronic inflammatory disorders. Stevia extract has multiple potential health benefits and may be a valuable addition to diabetes treatment plans and anti-aging therapies.

## Figures and Tables

**Table 1:** The % inhibition of AG and *Stevia* on protein glycation and aggregation in BSA + fructose system.

% Inhibition	Primary Stage		Intermediate Stage	Advanced Stage						
	Browning	Fructosamine	Carbonyl Content	Total AGEs	Individual AGEs				Congo Red	ThT
					Argpyrimidine	Vesperlysine	Pentosidine	Crossline		
AG	85	70	74	35	30	45	35	42	50	19
*Stevia*	71	67	64	81	82	81	80	70	47	31

**Table 2:** List of various AGEs’ excitation and emission wavelengths [66].

AGEs	Excitation Spectrum (nm)	Emission Spectrum (nm)
Argpyrimidine	320	380
Vesperlysine	350	405
Pentosidine	335	385
Crossline	380	440

## Data Availability

Not applicable.

## Sample Availability

Samples of the compounds not are available from the authors.

## Abbreviations

AG	Aminoguanidine
AGEs	Advanced Glycation End Products
BSA	Bovine serum albumin
DNPH	2,4-Dinitrophenylhydrazine
H_2_O_2_	Hydrogen Peroxide
HDF	Human Dermal Fibroblast
MG	Methylglyoxal
NaN_3_	Sodium azide
ROS	Reactive Oxygen Species
PEPCK	Phosphoenolpyruvate carboxykinase
ThT	Thioflavin T
mM	Milimolar
µL	Microliter
μM	Micromolar
